# Human Health Risk Assessment due to Global Warming – A Case Study of the Gulf Countries

**DOI:** 10.3390/ijerph5040204

**Published:** 2008-11-28

**Authors:** Tahir Husain, Junaid Rafi Chaudhary

**Affiliations:** Faculty of Engineering and Applied Science, Memorial University of Newfoundland, St. John’s, NL, A1B 3X5 Canada

**Keywords:** Climate change, Human health, Gulf countries

## Abstract

Accelerated global warming is predicted by the Intergovernmental Panel on Climatic Change (IPCC) due to increasing anthropogenic greenhouse gas emissions. The climate changes are anticipated to have a long-term impact on human health, marine and terrestrial ecosystems, water resources and vegetation. Due to rising sea levels, low lying coastal regions will be flooded, farmlands will be threatened and scarcity of fresh water resources will be aggravated. This will in turn cause increased human suffering in different parts of the world. Spread of disease vectors will contribute towards high mortality, along with the heat related deaths. Arid and hot climatic regions will face devastating effects risking survival of the fragile plant species, wild animals, and other desert ecosystems. The paper presents future changes in temperature, precipitation and humidity and their direct and indirect potential impacts on human health in the coastal regions of the Gulf countries including Yemen, Oman, United Arab Emirates, Qatar, and Bahrain. The analysis is based on the long-term changes in the values of temperature, precipitation and humidity as predicted by the global climatic simulation models under different scenarios of GHG emission levels. Monthly data on temperature, precipitation, and humidity were retrieved from IPCC databases for longitude 41.25°E to 61.875°E and latitude 9.278°N to 27.833°N. Using an average of 1970 to 2000 values as baseline, the changes in the humidity, temperature and precipitation were predicted for the period 2020 to 2050 and 2070 to 2099. Based on epidemiological studies on various diseases associated with the change in temperature, humidity and precipitation in arid and hot regions, empirical models were developed to assess human health risk in the Gulf region to predict elevated levels of diseases and mortality rates under different emission scenarios as developed by the IPCC. The preliminary assessment indicates increased mortality rates due to cardiovascular and respiratory illnesses, thermal stress, and increased frequency of infectious vector borne diseases in the region between 2070 and 2099.

## 1. Introduction

Exponential population growth, rapid industrialization, urbanization and transportation are causing enormous stress on energy consumption, which has resulted in the increased anthropogenic greenhouse gas emissions (GHG) such as carbon dioxide (CO_2_), methane (CH_4_) and nitrous oxide (NO_x_). The elevated levels of these greenhouse gases in the atmosphere are bound to cause considerable variations in daily, seasonal, inter-annual and decadal variabilities in our climate.

The historical meteorological records over the last few decades show a changing global climate due to human-induced emissions. IPCC projects a rise of 1.4 °C to 5.8 °C in the global temperature by the end of this century. It also predicts considerable rise in sea level, risking the inundation of the low lying coastal areas. Watson and McMichael [1] estimate that a one meter rise in the sea levels could cause Bangladesh to loose 20% of its land area and 33% of its present arable land.

Climatic changes will have increased direct and indirect risk to human health through different pathways and mechanisms. WHO estimates attribute more than 150,000 deaths with 5 million “disability–adjusted life years” (DALYs) due to diseases affected by the changing climate in the last three decades [2].

The climatic variations due to elevated temperatures, altered precipitation and humidity levels have the potential to cause adverse impacts on human health as well as on our ecological systems. The observed impacts will include increased morbidity and mortality due to thermal extremes, diarrhea, cholera, malnutrition, geographical and seasonal spread infectious vector and rodent borne diseases, and cardiovascular and respiratory illnesses. Apart from these direct effects, the indirect effects would cause increased frequency and severity of storms and flooding of the low lying coastal areas, droughts in some parts of the world, shortage of the fresh water resources and reduction in the agricultural yields. Psychological issues due to extreme weather events, natural disasters, limited food supplies, forced migration and dislocation at individual and community levels can lead to conflicts and hence will pose a major challenge for the authorities/ governments.

## 2. Purpose

The purpose of this study is to investigate and summarize the effects of climate changes and its impact on the human health in one of the vulnerable, arid and sandy region of the world, i.e. the Gulf countries. Although the region is strategically located along one of the world's most active shipping lanes and oil reserves along the Arabian Peninsula it has not been comprehensively studied for the future climate changes and their impacts on health as well as on natural ecological systems.

The present study encompasses coastal regions of the Gulf including countries like Yemen, Oman, United Arab Emirates, Qatar and Bahrain. The demographic situation of these countries is varied, with a temperate latitude location having extremely hot and dry climate.

The region already faces several environmental challenges including coastal degradation primarily due to frequent oil spillage, frequent sandstorms and dust-storms, sporadic droughts due to aridity and hot climate, very little cultivatable land, inadequate fresh water resources, and significantly increased dependence on the desalination facilities [3].

## 3. Method

The Special Report on Emissions Scenarios (SRES) developed by the IPCC defines a set of future emission scenarios based on greenhouse gas (GHG) and aerosol emissions for the 21^st^ century. The scenarios rely on well-defined assumptions about their driving forces regarding demographic nature, anticipated socio-economic and technological advancements in future based on projected energy consumption and population growth rates.

In order to develop possible anticipated scenarios for future predictions regarding climate changes, critical assumption of no-reduction in emissions was made. Several possible scenarios defined by the IPCC were considered and the two most likely and widely used scenarios were considered for this study as follows:

i) A-2, also known as “fragmented world scenario”, represents a world with unchecked population growth, considerable disparity in *per capita* income, self-dependence and local identities with regional based technological advancement.
ii) B-2, also known as “local sustainability scenario”, assumes a world with unchecked population growth, modest economical growth, slower but diverse technological development, emphasizing possible regional based solutions of socio-economic concerns.

Necessary monthly time series data for temperature, precipitation and humidity by the three global climate models was retrieved for the two scenarios based on the Third Assessment Report (TAR) from the United Nation’s Intergovernmental Panel on Climate Change (IPCC) data distribution centre “http://www.mad.zmaw.de/IPCC_DDC/html/SRES_TAR/index.html” [4]. The data in grib format (binary) was converted to numeral format by downloading software called “grib-converter” from “http://cera-www.dkrz.de/IPCC_DDC/GRIBGZIP.html”. Then using a computer program (coded in C++) we retrieved data for the latitude and longitude coordinates encompassing Yemen, Oman, United Arab Emirates, Qatar and Bahrain. The coordinate ranges in which all of the countries lie extend from longitude 41.25°E to 61.875°E and latitude 9.2779°N to 27.8334°N in the global data. The grid definitions of the three global climate models used for data collections are listed in Table 1. These models are briefly described as follows:

1. **Hadley Centre Climate Prediction and Research Model** (HADCM3): It is a coupled atmosphere-ocean GCM developed at the Hadley Centre. It has a stable control climatology and does not use flux adjustment. The atmospheric component of the model has 19 levels with a horizontal resolution of 2.5 degrees of latitude by 3.75 degrees of longitude, which produces a global grid of 96 × 73 grid cells.
2. **Canadian center for Climate Modeling and Analysis** (CCMa): This model has been used to produce ensemble climate change projections using the older IS92a forcing scenario, as well as the newer IPCC SRES A2 and B2 scenarios.
3. **National Center for Atmospheric Research** (NCAR): It is the latest in a series of global atmosphere models developed at NCAR for the weather and climate research communities. It is a dynamic-thermodynamic model that includes a sub-grid scale ice thickness distribution, energy conserving thermodynamics, and elastic-viscous-plastic (EVP) dynamics.

The Gulf region, comprising the abovementioned five countries, indicated considerable variations in predicted results for the three parameters over the next fifty year period (2020 to 2050) known as “S-1” and hundred year period (2070 to 2099) known as “S-2” compared with the baseline period (1970 to 2000). The predicted variations in temperature, precipitation and humidity by the three models under the fragmented world (A-2) and local sustainability (B-2) scenarios are shown in the table below:

**Table 2:** Data predicted for three parameters by models with selected scenarios.

**Parameter**	**Hadley(A-2)**	**Hadley (B-2)**	**CCCma(A-2)**	**CCCma(B-2)**	**NCAR (A-2)**	**NCAR (B-2)**
**Temperature**	°K	°K	°K	°K	°K	°K
**S-1**	0.94 to 1.23	0.78 to 1.05	0.79 to 1.09	0.67 to 0.94	0.40 to 0.66	0.31 to 0.55
**S-2**	2.58 to 2.93	1.93 to 2.47	2.47 to 3.37	1.55 to 2.16	1.33 to 1.80	NA
**Precipitation**	mm	mm	mm	mm	Mm	mm
**S-1**	−44.6 to 108.39	−12.7 to 144.3	−152.7 to 103.8	−197.2 to 61.2	−102.5 to 138.5	−0.12 to 145.07
**S-2**	3.0 to 485.90	16.9 to 530.4	−147.4 to 174.1	−126.0 to 113.6	−157.4 to 458.1	NA
**Relative**	%	%	g/kg	g/kg	g/kg	g/kg
**Humidity**						
**S-1**	−0.12 to 0.44	− 0.16 to 0.71	0.74 to 1.13	0.68 to 0.95	0.43 to 0.69	0.26 to 0.58
**S-2**	0.08 to 1.39	0.25 to 1.76	3.08 to 3.72	1.85 to 2.25	1.09 to 1.92	NA

## 4. Results

The predicted temperature, precipitation, and humidity levels by the three Global Climate Models vary considerably with regards to their ranges (as seen from the Table-2 above). They also have significantly inconsistent trends in their predictions for the different regions within the countries e.g. for years 2070 to 2099 Hadley model predicts average precipitation levels to increase in the entire region with considerable variations (+3.1 mm to +485 mm overall), and a lowest anticipated increase of about 50 mm. In contrast, the CCCma model predicts precipitation range of −147.4 mm to +174.1 with an anticipated shortfall in Yemen with the worst hit areas facing shortfall of >100 mm and Oman’s coastal regions from Salalah to Ra’sal expected to face a shortfall of >20 mm. CCCma however anticipates increased precipitation for Bahrain, Qatar, U.A.E and areas like Ibri in Oman. Similarly, the NCAR model predicts considerable precipitation variations range of −157.4 mm to 458.1 mm for the region, with areas like Aden, Tàizz and Sanna in Yemen and Salalah in Oman expected to receive higher precipitations, i.e. more than 200 mm. Bahrain, Qatar and U.A.E., along with Ibri, Muscat and Al Mudaybi in Oman are predicted to face negligible variations by this model, which also anticipates increased precipitation over other parts of Oman.

The predicted capabilities of these models with regards to other two parameters i.e. temperature and humidity over the same period show identical trends.

### 4.1. Health Impacts

Health impacts due to various pathways combined together as a result of climate change are calculated on the basis of the altered rates of specific diseases, and disability adjusted life years “DALY” (life years lost due to premature death and/or years lived with disability).

Burden of disease is calculated on the basis of available quantitative data and its correlation with specific disease incidence within population groups/regions based on the dose-response health impacts. Application of a similar approach based on ecosystem effects is quite complicated due to interactions with various casual pathways, about which there is not enough data available at this stage.

The health risk assessment is considered relative to future predictions in climate change due to increased GHG emissions with exposure to entire global population, but having varied impact depending on demographic factors and geographic locations. Most of the studies on health-impact of climate change have so far been empirical in nature i.e., relying either on statistical modeling techniques which involve direct link between climate variables and disease incidence, or biological modeling which rely on pathogens dynamics and climate effects. The influence of non-climatic factors like reduced immunity levels, malnutrition, drug resistance of the individuals have not been quantified and should be assessed with utmost care in other studies [5–7] as their contribution can be significant.

- 3% increase in death rates per 1 °C increase in temperature for all-cause mortality for the hot and arid regions where the temperature of the warmest months exceed 30 °C, including the above Gulf countries was reported by McMichael [7]. The threshold temperature for the estimation of the temperature-attributable mortality was 23 °C.
- Hajat *et al*. [8] studied mortality in London from January 1976 to December 1996 on the basis of time-series analysis and concluded that >97 percentile average temperature resulted in increased mortality rates by 3.34%, 2.47% and 4.23% for every 1 °C rise in temperature, longest duration, and highest temperature respectively.
- Significant increases in mortality rates was observed at elevated temperatures for all of the 27 world cities included in study conducted by Kalkstein and Smoyer [9] for different climate change scenarios, with or without acclimatization based on the model acquired from the synoptic air mass and mortality.
- Dessai [10], while studying temperature-mortality in Lisbon, Portugal, used empirical statistical model with input data from two regional climate models HadRM2 and PROMES, predicted the increase in mortality rates from 57-113% by 2020 and 97-225% by 2050.
- Based on measured and modeled CO_2_ concentrations of 550 ppm, 750 ppm and unmitigated emissions in the environment McMichael *et al*. [7] projects the relative risks of cardio-vascular diseases for all age groups for these scenarios with reference to the baseline scenario for the Eastern Mediterranean region (EMR-B) comprising Bahrain, Oman Qatar and United Arab Emirates to increase from 1.000, 1.001 and 1.001 during 2000 to 1.002, 1.002 and 1.003, respectively, for central estimates (adjusted biological adaptation) in the year 2030. In the EMR-D region comprising Yemen the average estimates for the entire region were also identical for central estimates. Whereas for higher estimates (no physiological or behavioral adaptation) the relative risk for EMR-B increased from 1.001, 1.001 and 1.002 to 1.004, 1.004 and 1.007 respectively while for EMR-D the noted increase range was from 1.001, 1.001 and 1.002 to 1.004, 1.005 and 1.007 between 2000 and 2030.

### 4.2. Malarial Risk

Climate changes are affecting the spatial and temporal spread of malarial vectors *Plasmodium falciparum* and rodent borne diseases. Harasawa *et al*. [11], based on the malarial AIM/impact model, predict that with 2°C increase in the temperature will cause considerable increase of malarial risk for the population living under endemic malarial conditions. It is reported that the entire population of UAE and Oman along with <50% of Yemen’s population are living in malarious regions. Though there will be no increase in the “High Risk” category of malarial diseases for the region, yet:

- “Low malarial risk” will remain unchanged for UAE (1.07 million affected population).
- “Low malarial risk” will reduce from 0.92 to 0.48 million affected people from Oman.
- “Low malarial risk” will rise markedly for Yemen by affecting larger population group i.e. from 1.08 to 2.38 million.

### 4.3. Projected mortality rates and DALYs

The World Health Organization (WHO) has categorized the globe into seven geographical regions. The Gulf countries lie in the Eastern Mediterranean Region (EMRO). Bahrain, Oman, Qatar and UAE lie in EMRO-B while only Yemen is in EMRO-D. WHO uses models to project mortality and DALY rates on the basis of regional divisions, sex, socio-economic development and their known relationship with cause-specific mortality rates. The EMRO has the third highest mortalities and DALY rates after south East Asian and African regions [6].

For the purpose of our investigation we took the central population estimates of the Gulf countries from the website (http://www.populationaction.org/) maintained by the Population Action International for year 2025 and 2050. Population estimates for the year 2100 were obtained by extrapolation. Based on the modeled results of McMichael *et al*. [7] and Hajat *et al*. [8], 1°C increase in average temperatures will result in a 3% increase in all-cause mortality, we estimate the excess all-cause mortality and DALY rates for the five Gulf countries to be as follows. The excess rates of all-cause mortality and DALY were determined on the basis of Hadley models prediction under A-2 emission scenario during 2070–2099, as summarized in Tables 3 and 4. Among all the scenarios presented in Table 2, Hadley A2 scenario was selected for further study because this scenario predicts high temperature increase and high ranges in precipitation and humidity changes due to global warming compared to other scenarios in Table 2.

As shown in Table 3 the mortality in the current year 2002 is compared with mortality in 2100 for adjusted population. Estimating the temperature increase in the selected gulf countries listed in Table 3 and using model results of increase in mortality due to excess temperature, the odd ratios were calculated which when multiplied with 2100 project all cause mortality will give projected temperature adjusted all cause mortality. The difference between this value and 2100 project all-cause mortality gives excess mortality per 100,000 population due to temperature increase as a result of global warming. Table 4 gives an estimate of disability-adjusted life years due to global warming effects. Similar procedure as discussed in Table 3 was adopted in such estimates.

## 5. Conclusions

Access to authenticated records of hospital admission and emergency room reporting/visits for illnesses such as thermal stresses and cardiovascular diseases should be available for detailed studies as they are a better source as compared to the empirical approaches. In-depth studies of the temperature–mortality relationship in the region are vital for precise estimation of population’s vulnerability and adaptation degree required due to climate changes to minimize mortality rates. Careful surveillance of climate-attributed infectious diseases should be carried out to monitor unusual disease incidence patterns as well as other risk indicators over time to be better equipped with disease specific measures to handle any possible pandemic situation.

The projected DALYs and mortality figures indicate that there will be significant increase in the disease incidence and affected population size while taking into account the rapid population growth rates, posing major challenge for the health services and national financial resources. The predicted data depicts Yemen to be most vulnerable nation already stressed because of its limited financial resources and higher population growth. It is thus emphasized that the adverse impact of climate change on human health in above Gulf countries necessitates emission reduction, well planned-housing and health-care infrastructures for future. Improved intervention programs capable of reducing population’s vulnerability towards direct and indirect adverse impacts should be initiated. Measures should also be taken to improve social and behavioral adaptation at individual, community and national levels with regards to the basic improvements in social structure and minimization of inequalities within population groups. Adapting necessary mitigative strategies can give health benefits in short term, but developing effective system of regionally based targeted public health intervention strategies is imperative for minimizing the climate relate health–risks.

## Figures and Tables

**Figure 1:**
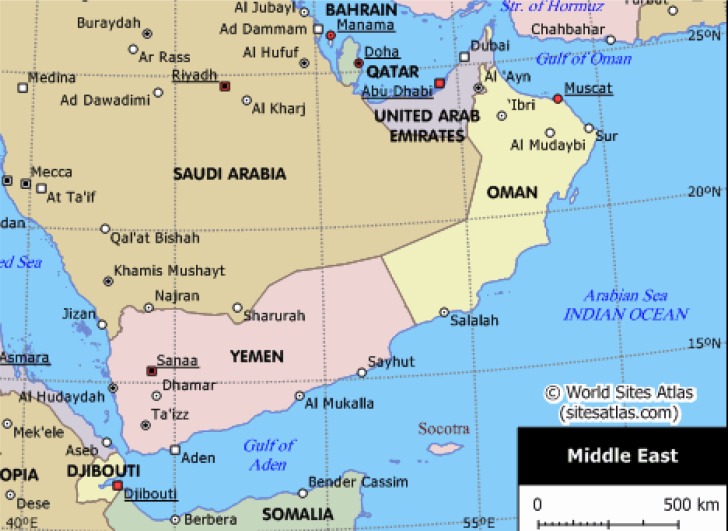
The Gulf region countries with their geographical co-ordinates.

**Table 1:** Global Climate models used for data collection with there grid definitions.

**Centre**	**Model**	**Longitude**	**Latitude**
Hadley	*HadCM3*	41.25 - 60.00 E	12.50 - 27.50 N
CCCma	*CGCM2*	41.25 - 60.00 E	9.2779 - 27.83 N
NCAR	*NCAR-PCM*	42.1875 - 61.875 E	12.55776 - 26.51 N

**Table 3:** Projected all-cause mortality.

**Country**	**2002 Population (`000)**	**2002 all-cause mortality**	**2100 extrapolated population est. (`000)**	**2100 Projected all-cause mortality**	**Hadley’s Projected temp. (°K) increase**	**Odd Ratio**	**Projected temp. adj. all-cause mortality**	***Excess mortality due to temp.***
Bahrain	709	322.1	1,115	506.6	2.55–2.61	1.0774	545.8	***39.2***
Oman	2,768	301.5	30,548	3327.4	2.62–2.82	1.0834	3604.9	***277.5***
Qatar	601	247.2	1,044	429.4	2.57–2.82	1.081	464.2	***34.8***
UAE	2937	303.7	4,540	484.9	2.57–2.86	1.0816	524.5	***39.6***
Yemen	19,315	887.0	145,746	6693.1	2.64–2.93	1.0837	7253.3	***560.2***

**Table 4:** Projected DALYs/100.000 population.

**Country**	**2002 Population (`000)**	**2002 all-cause DALY /100,000**	**2100 extrapolated population est. (`000)**	**2100 Projected all-cause DALY**	**Hadley’s Projected temp. (°K) increase**	**Odd Ratio**	**Projected temp. adj./ all-cause DALY**	***Excess DALY due to temp.***
Bahrain	709	11,726	1,115	18,440	2.55–2.61	1.0774	19,868	***1,428***
Oman	2,768	13,121	3,0548	144,805	2.62–2.82	1.0834	156,881	***12,076***
Qatar	601	11,742	1,044	20,397	2.57–2.82	1.081	22,049	***1,652***
UAE	2,937	14,067	4,540	21,744	2.57–2.86	1.0816	23,519	***1,775***
Yemen	19,315	35,932	145,746	271,133	2.64–2.93	1.0837	293,827	***22,694***
